# Biocloud: Cloud Computing for Biological, Genomics, and Drug Design

**DOI:** 10.1155/2013/909470

**Published:** 2013-10-02

**Authors:** Ching-Hsien Hsu, Chun-Yuan Lin, Ming Ouyang, Yi Ke Guo

**Affiliations:** ^1^Department of Computer Science and Information Engineering, Chung Hua University, Hsinchu 30012, Taiwan; ^2^Department of Computer Science and Information Engineering, Chang Gung University, Taoyuan 333, Taiwan; ^3^Department of Computer Engineering & Computer Science, University of Louisville, Louisville, KY 40292, USA; ^4^Department of Computing, Imperial College London, London SW7 2AZ, UK

## 1. Introduction

Cloud computing has emerged rapidly as an exciting new paradigm that offers a challenging model of computing and services. Leveraging cloud computing technology, bioinformatics tools can be made available as services to anyone, anywhere, and through any device. The use of large biodatasets, its highly demanding algorithms, and the hardware for sudden computational resources makes large-scale biodata analysis an attractive test case for cloud computing.

This special issue aims to foster the dissemination of high quality research in any new idea, method, theory, and technique related to cloud computing and bioinformatics and to showcase the most recent developments and research in cloud computing for biological, genomics, and drug design, considering genomics and drug design on the cloud, biological tools on the cloud, biodatabase on the cloud, cloud-based biocomputing, and all kinds of successful applications. The research papers selected for this special issue represent recent progresses in the aspects, including theoretical studies, practical applications, new analysis and modeling technology, programming methodologies, and experimental prototypes. All of these papers not only provide novel ideas and state-of-the-art techniques in the field but also stimulate future research in the biocloud environments.

## 2. Cloud-Based Biological Service

Large-scale scientific experiments have an ever increasing demand for High Performance Computing (HPC) resources. The paper by R. De Paris et al. “*wFReDoW: a cloud-based web environment to handle molecular docking simulations of a fully flexible receptor model*” proposes a cloud-based web environment, called web Flexible Receptor Docking Workflow (wFReDoW), which reduces the CPU time in the molecular docking simulations of FFR models to small molecules. It is based on the new workflow data pattern called Self-adaptive Multiple Instances (P-SaMI) and on a middleware built on Amazon EC2 instances. P-SaMI reduces the number of molecular docking simulations while the middleware speeds up the docking experiments using a High Performance Computing (HPC) environment on the cloud. The experimental results show a reduction in the total elapsed time of docking experiments and the quality of the new reduced receptor models produced by discarding the nonpromising conformations from an FFR model ruled by the P-SaMI data pattern.

On the other hand, as bioinformatics is embracing cloud computing, the paper by L. Kaján et al. entitled “*Cloud prediction of protein structure and function with PredictProtein for Debian*” reports the release of PredictProtein for the Debian operating system and derivatives, such as Ubuntu, Bio-Linux, and Cloud BioLinux. The PredictProtein suite is available as a standard set of open source Debian packages. The release covers the most popular prediction methods from the Rost Lab, including methods for the prediction of secondary structure and solvent accessibility (profphd), nuclear localization signals (predictnls), and intrinsically disordered regions (norsnet). The authors also present two case studies that successfully utilize PredictProtein packages for high performance computing in the cloud.

## 3. High-Performance Biological Computing

Although the computer science technologies can be used to reduce the costs of the pharmaceutical research, the computation time of the structure-based protein-ligand docking prediction is still unsatisfied until now. The paper by J.-L. Chen et al. entitled “*A high performance cloud-based protein-ligand docking prediction algorithm*” presents a novel docking prediction algorithm to accelerate the docking prediction. The proposed algorithm works by leveraging two high-performance operators: (1) the novel migration (information exchange) operator is designed specially for cloud-based environments to reduce the computation time; (2) the efficient operator is aimed at filtering out the worse search directions. The simulation results illustrate that the proposed method outperforms the other docking algorithms compared in this paper in terms of both the computation time and the quality of the end result.

The proteome-wide analysis of protein-ligand binding sites and their interactions with ligands is potentially an important source of information in structure-based drug design and in understanding ligand cross-reactivity and toxicity. The paper by C.-L. Hung and G.-J. Hua entitled “*Cloud computing for protein-ligand binding site comparison*” develops a cloud computing service, called Cloud-PLBS, combining SMAP and Hadoop framework, and it is deployed on a virtualization cloud computing platform. Cloud-PLBS takes advantage of the MapReduce paradigm as means of management and parallelizing tool under massive number of protein-ligand binding site pairs compared under the experiment. Cloud-PLBS provides both a web portal and scalability for biologists to address a wide range of compute intense questions in biology and drug discovery. The performance experiment shows that it is desirable for molecular biologists to investigate the protein structure and function analysis under reasonable time constraints by using our cloud service.

An understanding of the activities of enzymes could help to elucidate the metabolic pathways of thousands of chemical reactions that are catalyzed by enzymes in living systems. The paper by C.-C. Huang et al. entitled “*Enzyme reaction annotation using cloud techniques*” proposes the enzyme reaction prediction (ERP) method as a novel tool to deduce enzyme reactions from domain architecture. We used several frequency relationships between architectures and reactions to enhance the annotation rates for single and multiple catalyzed reactions. The deluge of information which arose from high-throughput techniques in the postgenomic era has improved our understanding of biological data, although it presents obstacles in the data-processing stage. The high computational capacity provided by cloud computing has resulted in an exponential growth in the volume of incoming data. Cloud services also relieve the requirement for large-scale memory space required by this approach to analyze enzyme kinetic data.

## 4. Big Data Intelligence

The rate of accumulation of biomolecular data is increasing astonishingly. This information explosion is being driven by the development of low-cost, high-throughput experimental technologies in genomics, proteomics, molecular imaging, amongst others. Success in the life sciences will depend on our ability to rationally interpret these large-scale, high-dimensional data sets into clinically understandable and useful information, which in turn requires us to adopt advances in informatics. The paper by J. Chen et al. entitled “*Translational biomedical informatics in the cloud: present and future*” demonstrates the utility and promise of cloud computing for tackling the big data problems. The authors outline their vision that cloud computing could be an enabling tool to facilitate translational bioinformatics research. Biomedical cloud, given the proper architecture, could integrate all the petabytes of available biomedical informatics data in one place and process them on a continuous basis. In this way, we would continuously observe the connections between genotypic profiles and phenotypic data. We can envision that the cloud-supported translational bioinformatics endeavours will promote faster breakthroughs in the diagnosis, prognosis, and treatment of human disease.

Based on the concepts of resources on demand and pay as you go, scientists with no or limited infrastructure can have access to scalable and cost-effective computational resources. However, the large size of next generation sequencing (NGS) data causes significant data transfer latency from the client's site to the cloud, which presents a bottleneck for using cloud computing services. The paper by S. A. Issa et al. entitled “*Streaming support for data intensive cloud based sequence analysis*” provides a streaming-based scheme to overcome this problem, where the NGS data is processed while being transferred to the cloud. The proposed scheme targets the wide class of NGS data analysis tasks, where the NGS sequences can be processed independently from one another. This study also provides the elastream package that supports the use of this scheme with individual analysis programs or with workflow systems. The experiments presented in this paper show that the proposed solution mitigates the effect of data transfer latency and saves both time and cost of computation.

## 5. GPU Technologies

With the endeavor to narrow performance overhead, the virtualization technology expands its coverage from cloud computing to high performance computing such as biological computation. Recently, biological applications start to be re-implemented into the applications which exploit many cores of GPUs for better computation performance. The paper by H. Jo et al. entitled “*Exploiting GPUs in virtual machine for BioCloud*” proposes a BioCloud system architecture that enables VMs to use GPUs in cloud environment. The proposed system exploits the pass-through mode of PCI express (PCI-E) channel. By making each VM to be able to access underlying GPUs directly, applications can show almost the same performance as when those are in native environment. The proposed scheme multiplexes GPUs by using hot plug-in/out device features of PCI-E channel. By adding or removing GPUs in each VM in on-demand manner, VMs in the same physical host can time-share their GPUs. The performance results showed that this prototype is highly effective for biological GPU applications in cloud environment.

The Smith-Waterman (SW) algorithm searches for a sequence database to identify the similarities between a query sequence and subject sequences. However, this algorithm is prohibitively high in terms of time and space complexity. The paper by S.-T. Lee et al. entitled “*GPU-based cloud service for Smith-Waterman algorithm using frequency distance filtration scheme*” presents a novel Smith-Waterman algorithm with a frequency-based filtration method on GPUs rather than merely accelerating the comparisons yet expending computational resources to handle such unnecessary comparisons. A user friendly interface is also designed for potential cloud server applications with GPUs. Experimental results indicate that reducing unnecessary sequence alignments can improve the computational time by up to 41%.

## 6. Conclusions

All of the above papers address either big data intelligence issues in cloud or cloud-based biological service or propose novel application models in the various cloud and ubiquitous fields. They also trigger further related research and technology improvements in application of Biological computing. Honorably, this special issue serves as a landmark source for education, information, and reference to professors, researchers, and graduate students interested in updating their knowledge about or active in biological computing, biocloud services and management, and novel application models for bioCloud services and computing systems.

